# Innate immune recognition and activation during HIV infection

**DOI:** 10.1186/1742-4690-7-54

**Published:** 2010-06-22

**Authors:** Trine H Mogensen, Jesper Melchjorsen, Carsten S Larsen, Søren R Paludan

**Affiliations:** 1 Department of Infectious Diseases, Aarhus University Hospital, Skejby, DK-8200, Aarhus N, Denmark; 2 Department of Medical Microbiology and Immunology, Aarhus University, DK-8000 Aarhus C, Denmark

## Abstract

The pathogenesis of HIV infection, and in particular the development of immunodeficiency, remains incompletely understood. Whichever intricate molecular mechanisms are at play between HIV and the host, it is evident that the organism is incapable of restricting and eradicating the invading pathogen. Both innate and adaptive immune responses are raised, but they appear to be insufficient or too late to eliminate the virus. Moreover, the picture is complicated by the fact that the very same cells and responses aimed at eliminating the virus seem to play deleterious roles by driving ongoing immune activation and progressive immunodeficiency. Whereas much knowledge exists on the role of adaptive immunity during HIV infection, it has only recently been appreciated that the innate immune response also plays an important part in HIV pathogenesis. In this review, we present current knowledge on innate immune recognition and activation during HIV infection based on studies in cell culture, non-human primates, and HIV-infected individuals, and discuss the implications for the understanding of HIV immunopathogenesis.

## Introduction and key questions in HIV pathogenesis

The natural history of HIV infection is characterized by an acute phase with very high circulating levels of virus and a rapid decline in CD4+ T cells [1,2]. Despite a strong immune response resulting in decreasing viral load and increasing numbers of circulating virus-specific CD4+ T cells following the acute phase, the host is not capable of clearing the infection [3,4]. This allows HIV to establish life-long latency and chronic infection with progressive fatal immunodeficiency if left untreated. In a paradoxical manner, HIV-induced immunodeficiency is not dominated by paresis and inactivity of the immune system, but rather by chronic immune activation and high cell turnover, apoptosis, and activation-induced cell death [4-6]. Although it is widely accepted in the field that persistent immune activation plays a central part in driving immunopathogenesis and progression to AIDS, the fundamental determinants of progressive cell loss and functional immune deficiency in HIV infection remain unexplained. How does acute HIV infection lead to depletion of cells in gut associated lymphoid tissue (GALT) and irreversible damage to the host immune system? Which molecular mechanisms may underlie the chronic immune activation eventually causing progressive immune exhaustion and profound immunodeficiency? These are central questions in the understanding of the pathogenesis of HIV infection, which remain unanswered despite intense research in this area since the discovery of HIV more than 25 years ago [7,8]. HIV targets central players of the immune system, including cells of the mononuclear lineage, such as T cells, monocytes, and macrophages, but whereas the role of the adaptive immune response has been extensively studied [4], much less knowledge exists regarding the role of innate immune recognition and inflammation during HIV infection.

## Immunopathogenesis

### Acute HIV infection

Acute or primary HIV infection is defined as the first period of infection from the detection of HIV RNA until the formation of HIV-specific antibodies 3-4 weeks after infection [1]. Following sexual transmission of HIV, the virus first replicates locally in the vaginal or rectal mucosa, and this early stage before detectable viral RNA in plasma is termed the eclipse phase. Molecular analyses of subjects with acute HIV infection have indicated that productive infection arises from a single infectious virus [9,10], and other studies suggest that the first cells to be infected in the mucosa are resident memory T cells expressing CD4 and CCR5 [11,12]. Already at this early point of infection, innate immune activation may contribute by recruiting granulocytes, macrophages, and lymphocytes, the latter two of which are cellular targets of the virus. Virus or virus-infected cells then reach the draining lymph nodes, where activated CD4+CCR5+ T cells are encountered and represent targets for further infection. In this process, virus particles are bound by dendritic cells (DC)s through the C-type lectin receptor (CLR) DC-SIGN, and also by B lymphocytes through the complement receptor CD21, thereby augmenting viral spread by carrying virus to activated T cells [13,14]. This allows the virus to replicate and disseminate to secondary lymphoid tissue throughout the organism, with a particular predilection for GALT, where activated CD4+CCR5+ effector memory T cells are present at high levels [15].

Studies in SIV models and HIV-infected individuals have documented that acute SIV/HIV infection is accompanied by a massive depletion of CD4+ memory T cells, primarily in mucosal tissue, which may be explained by the high expression of the viral co-receptor CCR5 and the relatively activated state of mucosal CD4+ T cells [15-19]. In later studies, it has been demonstrated that as much as 60% of CD4+ memory T cells throughout the organism, including blood, lymph nodes and GALT are infected by SIV, and that the majority of these cells disappear within few days [20]. Importantly, the depletion of CD4+ memory T cells is not restricted to T cells of mucosal origin, although quantitatively most cells are lost from the mucosa, because the greatest number of T cells is resident in this location [20]. As to the cellular mechanism underlying this massive CD4+ T cell depletion, another study in SIV-infected rhesus macaques found that SIV exploits a large resident population of CD4+ memory T cells to produce high levels of virus that both directly, through lytic infection, and indirectly, through Fas-mediated apoptosis of infected and uninfected cells, deplete the majority of CD4+ T cells in GALT within the first 3 weeks of infection [21]. However, acute infection does not efficiently target naïve and resting central memory T cells, which do not express CCR5, leaving the regenerative potential of these T cell populations relatively intact at this stage [4].

Plasma viraemia increases to reach a peak after 21-28 days of infection together with depressed peripheral CD4+ T cell numbers. Whereas the amount of circulating T cells subsequently return close to normal, CD4+ T cell numbers in the GALT remain severely reduced [18,22]. Thus, acute HIV infection is accompanied by a selective and dramatic depletion of CD4+CCR5+ memory T cells predominantly from mucosal surfaces. This loss is largely irreversible and has profound immunological consequences, eventually manifesting as failure of the host immune defences and progression to AIDS later during infection [23].

**Figure 1:**
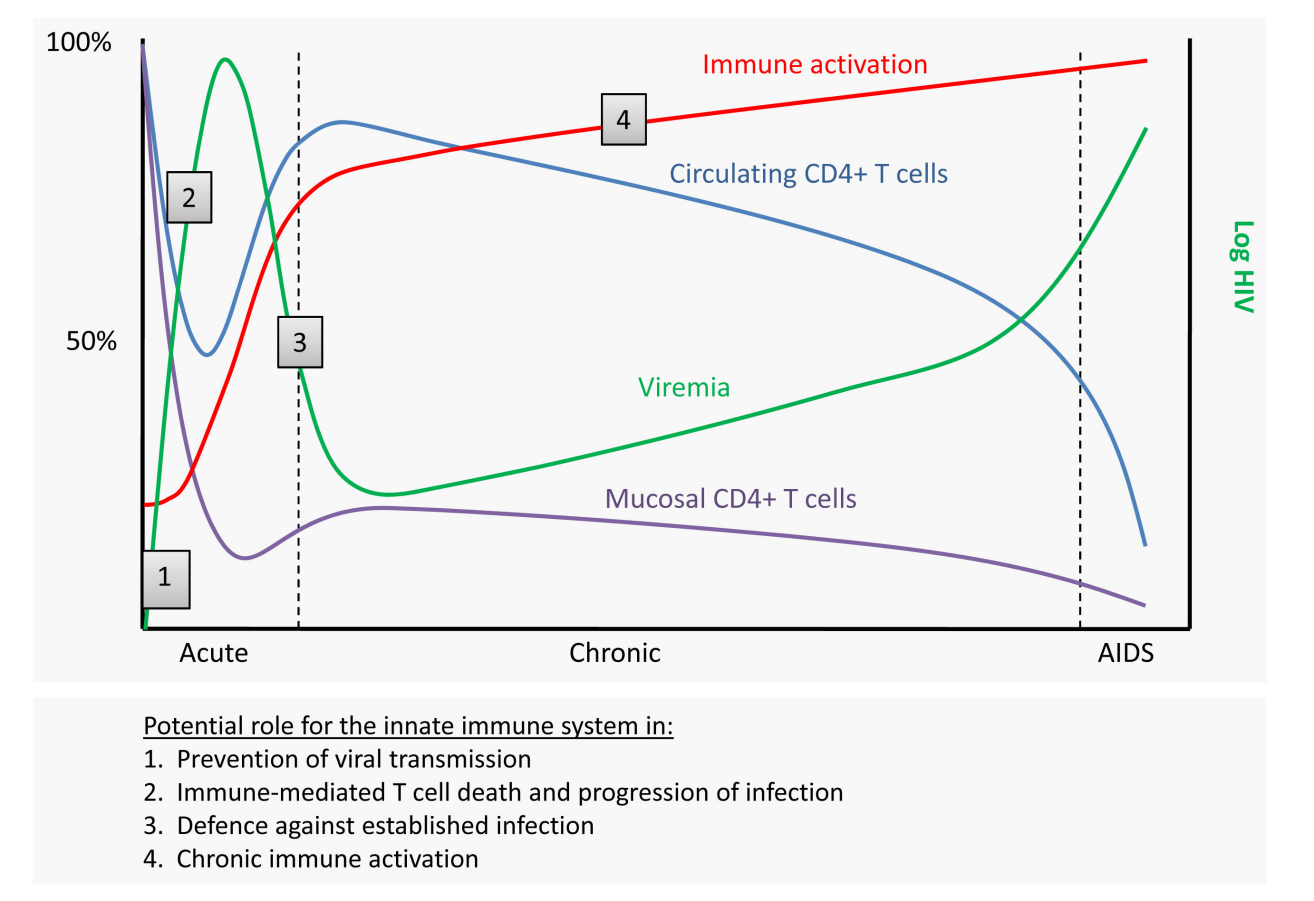
**Potential roles of the innate immune system during HIV infection**. (1) Following exposure at mucosal surfaces, HIV is transmitted with very low transmission efficiency, indicating that innate antiviral mechanisms are operative to prevent establishment of infection. (2) The early inflammatory response leads to recruitment and activation of various leukocytes, some of which serve as target cells for *de novo *HIV infection. (3) After acute infection, circulating viral load is generally decreased to a low level. This is mediated by the adaptive immune response, which is activated through processes driven by the innate immune response. Moreover, direct innate antiviral mechanisms contribute to control of virus replication during the chronic phase. (4) Persistent immune activation during chronic HIV infection involves activities stimulated by HIV-derived or opportunistic PAMPs through PRRs.

At the time of peak viraemia, patients may develop symptoms of the acute retroviral syndrome, including influenza-like illness with fever, sore throat, lymphadenopathy, and exanthema [24]. However, viral reservoirs have already been established in cells with slower rate of decay than T cells, implying that the virus cannot be eliminated by highly active antiretroviral treatment (HAART) within the life time of the patient [25]. Eventually, the viral load decreases over 12-20 weeks to reach a stable viral set point [26], and this initiates a more chronic phase of the infection. In primate models of SIV infection, it has been demonstrated that in the absence of CD8+ T cells, virus levels do not decline from peak viraemia for a prolonged period, implicating that CD8+ T cells play a crucial role in suppressing SIV replication [27,28]. This is supported by studies in HIV-infected individuals demonstrating major oligoclonal expansions of CD8+ T cells during acute HIV infection as well as associations between virus-specific CD8+ T cell activity and control of viraemia [29,30]. Therefore, it has been anticipated that CD8+ T cell-mediated control of viraemia is mediated by cytotoxic killing of productively infected cells [27,30]. However, more recent reports have challenged this assumption by demonstrating that CD8+ T cell suppression is not mediated by cytotoxic clearance of infected cells, and that the life span of infected cells is not decreased, indicating that the role of CD8+ T cells may be much more complex [27,31-33]. The central immunological parameters in the natural history of HIV infection is depicted in Figure 1, which also illustrates how the innate immune system plays a part in early restriction of the virus and shaping of the adaptive immune response, but at the same time participates in the establishment and spread of infection. This is discussed in details later in this review.

### Chronic HIV infection

Despite the return of circulating CD4+ T cells to near normal levels and the infection being largely asymptomatic for extended time periods in the majority of patients, it is now well established that massive immune activation and an accelerated cell turnover takes place during chronic HIV infection [34,35] (Figure 1). This apparent state of basal immune hyper-activation in the infected host is evidenced by increased expression of activation markers, such as CD38, HLA-DR and Ki67, of which CD38 is considered the most reliable surrogate marker for immune activation, disease progression to AIDS, and death [36]. In the gut, naïve and central memory T cells are supplied, but these cells are short-lived and only partially substitute for the CD4+ effector memory T cells depleted during the acute phase of infection [4,37]. In contrast, by stimulating this tremendous naïve and central memory CD4+ T cell repertoire that is programmed to generate additional CCR5 expressing targets, the virus creates new sources of infection and avoids the consequences of target cell depletion [23].

The profound immunological damage to the gastrointestinal tract leads to breaks in the mucosal barrier allowing translocation of microbial products, including bacterial lipopolysaccharide (LPS), into the circulation. A seminal study by Brenchley and colleagues demonstrated bacterial translocation during HIV infection and correlated plasma LPS levels with immune activation [38]. Bacterial translocation may therefore represent a crucial event in persistent immune activation, although it is probably not the only source of the microbial burden responsible for chronic immune activation (Figure 1). Intriguingly, HIV itself may also be a central player in the process due to viral constituents, such as glycoprotein (gp)120 and nef, or viral nucleic acids produced during viral replication, subsequently resulting in activation of proinflammatory cytokines and type I interferon (IFN), including IFN-α and IFN-β [1,4,39]. These aspects are discussed in further detail below. The ultimate consequence of immune activation is depletion of CD4+ T cells by different mechanisms, including a decrease in CD4+ and CD8+ T cell half-life, abnormal T cell trafficking, clonal exhaustion of T cells, and drainage of memory T cell pools [40-42]. Intriguingly, during chronic HIV infection only a minority of activated T cells are HIV-infected or HIV specific [23,42]. Nevertheless, CD4+ T cells are profoundly depleted and replaced by short-lived T cells with a more limited regenerative potential [4]. Another important factor is the accelerated viral evolution at this stage, provided by an excessively high viral mutation rate and alteration in cellular tropism, resulting in progression from a pool of CCR5-trophic to dual trophic or dominantly CXCR4 trophic strains with increased virulence and broader target cell trophism [4]. In addition, damage to lymphoid tissue results in thymic dysfunction, transforming growth factor-β-dependent fibrosis and alterations in lymphoid follicle architecture [41,43]. HIV infection also profoundly affects blood and tissue B cells by inducing early class switching in polyclonal B cells, massive B cell apoptosis, and loss of germinal centers in lymphoid tissue [44,45]. Although the profound damage to the adaptive immune system dominates, it has been increasingly appreciated that most other parts of the immune system, particularly innate immune defences, are also significantly dysregulated [1].

Finally, important questions regarding the immunopathogenesis of HIV infection may be learned from the study of infection in natural hosts, or potentially from HIV-infected humanized mouse models [46]. Intriguingly, simian immunodeficiency virus (SIV) infection in sooty mangabeys that represent natural hosts of SIV leads to high viral load but only very modest immune activation [47]. In contrast, SIV infection in rhesus macaques, which are not natural hosts and therefore mount a strong immune response, resemble human HIV infection with production of inflammatory mediators at the expense of the development of immunodeficiency [47,48]. Such findings support the idea that immune activation is primarily disadvantageous to the host and a major driving force for immune exhaustion during human HIV infection. This is in part due to enhanced activation of CD4+ T cells resulting in increased targets for HIV infection, but also a result of the undesirable effects of generalized immune activation more globally within the immune system. These observations therefore raise the question, whether HIV infection might be less detrimental for the immune system, had the immune response to the virus been less powerful.

## Innate immunity and pattern recognition receptors

Since one of the fundamental characteristics of HIV pathogenesis is the failure of the immune system to recognize, control, and eliminate the virus, much focus has been on early events following viral infection. The innate immune system constitutes the first line of defence against invading pathogens and is based on epithelial barriers, the complement system, and cells with phagocytotic and antigen presenting properties, such as granulocytes, macrophages, and DCs respectively [49,50].

Pattern recognition receptors (PRR)s have been assigned a central role in innate immune defences due to their ability to recognize evolutionarily conserved structures on pathogens, termed pathogen-associated molecular patterns (PAMP)s. A limited number of germ-line encoded receptors are responsible for triggering an innate immune response following the encounter with PAMPs, which are characterized by being invariant among entire classes of pathogens, essential for survival of the pathogen, and distinguishable from self [51]. Among PRRs, the family of Toll-like receptors (TLR)s have been studied most extensively. TLRs are membrane-bound receptors with 10 different TLRs identified in humans. TLR1, 2, 4, 5, 6, and 10 are expressed at the cell surface and mainly recognize hydrophobic molecules unique to microbes and not produced by the host. In contrast, TLR3, 7, 8, and 9 are located almost exclusively in endosomal compartments and are specialized in recognition of nucleic acids. Hence, non-self discrimination is provided primarily by the exclusive localization of the ligands rather than solely based on a unique molecular structure different from that of the host [50]. For example, TLR2 recognizes lipoteichoic acids of gram-positive bacteria, whereas TLR4 is activated by LPS of gram-negative bacteria, and additionally, TLR2 and TLR4 are involved in the response to certain viral surface glycoproteins [52-54]. However, viral recognition is primarily mediated by TLR9 recognizing DNA, as well as by TLRs 7/8, and 3 sensing single-stranded (ss) RNA and double-stranded (ds) RNA, respectively [55-59]. In addition, CLRs, such as DC-SIGN, Dectin-1, and mannose receptor, have emerged as cell surface PRRs that play important roles in induction of immune responses against various pathogens [60]. DC-SIGN in particular, has been attributed essential roles as an adhesion receptor, in mediating interactions between DCs and T cells, and as a PRR inducing specific immune responses [13,61].

**Figure 2:**
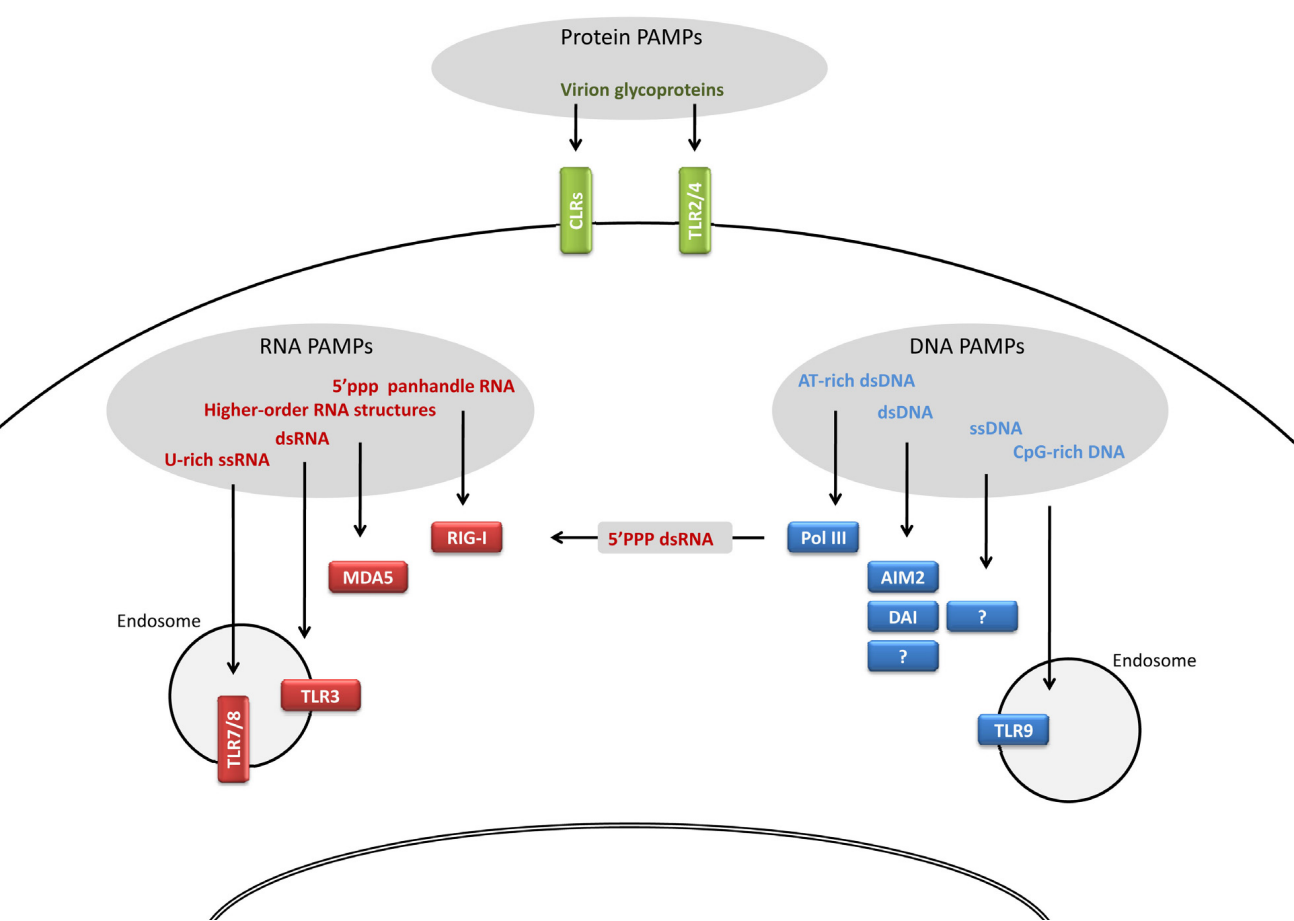
**Viral PAMPs and related cellular PRRs**. Viral glycoproteins may be recognized by TLR2/4 or CLRs on the cell surface. In the intracellular environment, various viral RNA and DNA structures are recognized by nucleotide sensors localized in endosomes or in the cytoplasm. It remains unknown whether nuclear PRRs exist able to recognize viral PAMPs in the nucleus.

Since microbial material is not exclusively present extracellularly or within endosomes, alternative cytosolic PRRs exist. The retinoid acid-inducible gene (RIG)-like receptors (RLR)s, RIG-I and MDA5, are RNA helicases that play a pivotal role in sensing of cytoplasmic RNA [62,63]. Studies have suggested differential roles of these helicases, with RIG-I being responsible for recognizing short dsRNA and 5'triphosphorylated panhandle RNA, whereas MDA5 responds to long dsRNA and higher order RNA structures [64-68]. Finally, cytosolic DNA receptors have been identified more recently and are the subject of much research interest in the field. The DNA receptors AIM2 and DAI respond to most types of dsDNA, in contrast to polymerase III-dependent responses that are restricted to AT-rich dsDNA [51,69-71]. Furthermore, a receptor for ssDNA may exist but has not presently been identified [72]. Figure 2 illustrates different classes of viral PAMPs and related PRRs on the cell surface and in the intracellular environment.

**Figure 3:**
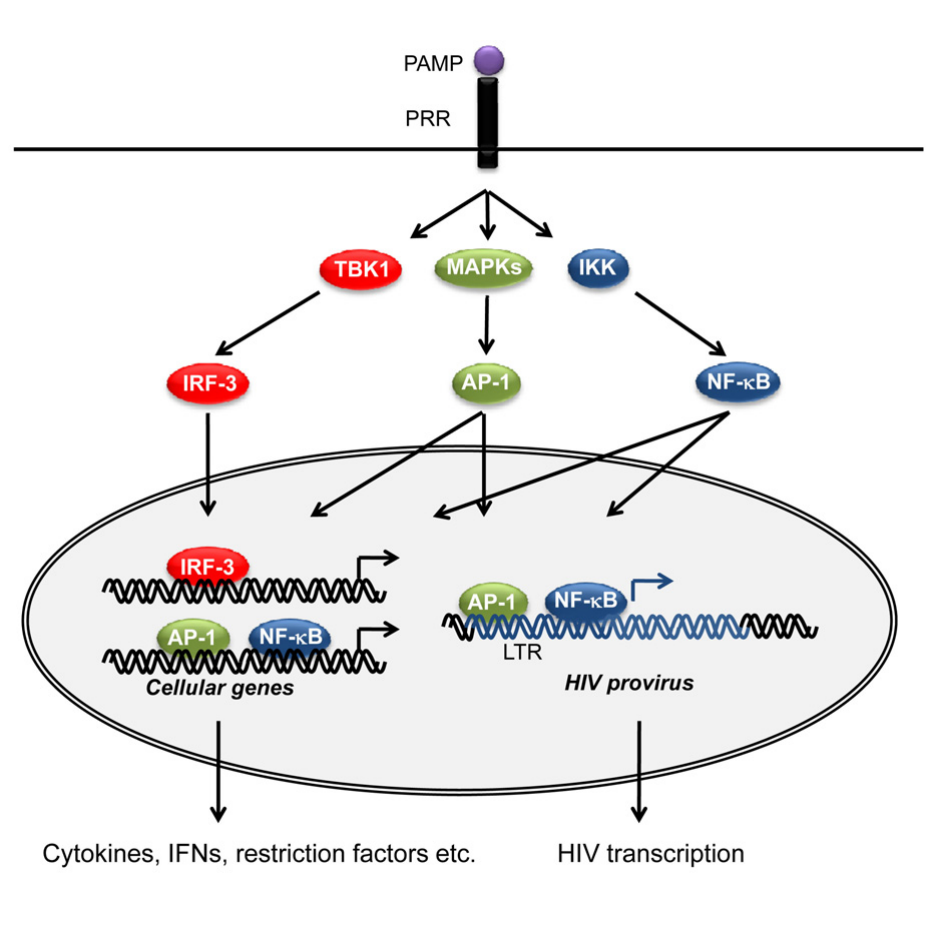
**Principles in PRR signalling and transcription of cellular genes and HIV provirus**. Sensing of microbial PAMPs by PRRs stimulates intracellular signalling pathways, leading to activation of transcription factors, notably NF-κB, IRF-1, and AP-1. These transcription factors bind to specific sequences present in gene promoter regions and activate transcription of antiviral and inflammatory genes. Importantly, NF-κB and AP-1 also activate transcription of the HIV provirus through binding to the corresponding elements in the HIV LTR to induce viral replication. TBK, TANK-binding kinase. IKK, IκB kinase.

Overall, ligand engagement of PRRs leads to activation of a proinflammatory and antimicrobial response by triggering signal transduction pathways involving the transcription factors nuclear factor (NF)-κB and IFN regulatory factors (IRF) 3/7 as well as mitogen-activated protein kinase (MAPK) pathways, ultimately resulting in the production of cytokines, chemokines, cell adhesion molecules and antiviral type I IFN. This is depicted in Figure 3. Some degree of specificity and selectivity is conferred by complex differences in the response depending on cell type, timing and localization. For instance, only a subset of TLRs, including TLRs 3, 7/8, 9, and to a lesser extent TLR4, can induce IFN due to their selective activation of IRFs [50,73]. CLR-induced intracellular pathways, which involve activation of the kinase Raf-1, essentially modulate the responses of other PRRs but also exert functions independently from other PRRs [60]. Importantly, innate immune activation is required for the subsequent activation and shaping of adaptive immunity, for instance by enhancing antigen presentation, by promoting DC recruitment and maturation, and finally by providing signals involved in DC-mediated CD4+ T cell polarization and priming [74].

## Candidate PAMPs generated during the HIV life cycle

When considering how HIV may possibly be recognized by the innate immune system, it seems logical to contemplate the possible PAMPs that are part of the HIV particle or generated during different phases of the viral life cycle. Being a member of the retroviridae family (lentivirus subfamily), HIV is a spherical enveloped RNA virus with a diameter of roughly 100 nm. The envelope contains viral glycoproteins and encloses a cone-shaped capsid containing two identical copies of the positive ssRNA genome of 10 kilobases together with several copies of reverse transcriptase (RT), integrase, additional viral proteins and two cellular tRNAs [75]. The viral genome contains three major structural genes, including gag, pol, and env, as well as six regulatory genes, namely vif, vpr, tat, rev, vpu, and nef. At each end of the genome are long-terminal repeat (LTR) sequences that contain promoters, enhancers, and other gene sequences required for binding of different cellular (or viral) transcription factors, such as NF-κB, Nuclear factor of activated T-cells, and activator protein (AP)-1, involved in viral replication [75] (Figure 3). Similar to cellular mRNA, the viral genome has a 5' cap and is poly-adenylated at the 3' end.

**Figure 4:**
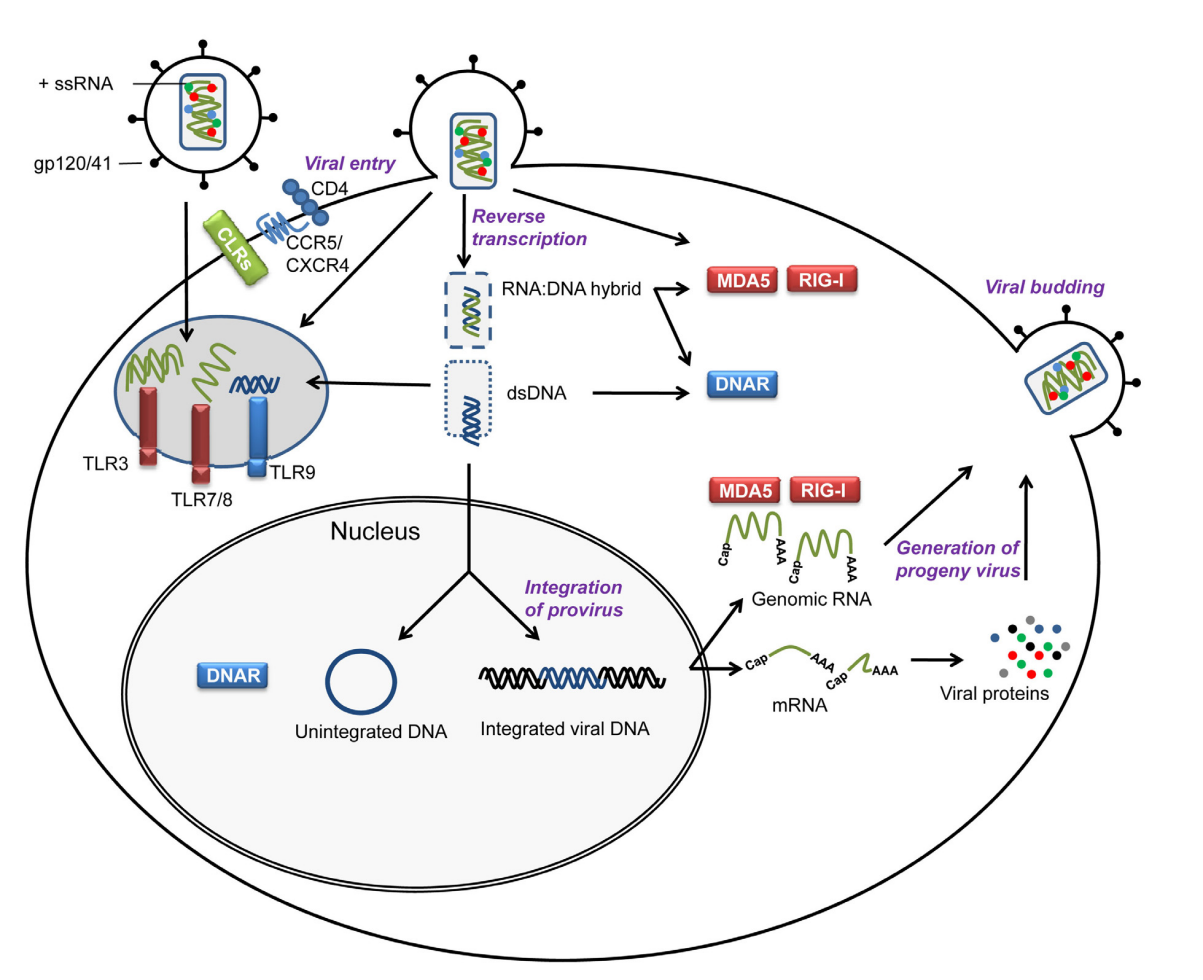
**Theoretical possibilities for innate immune recognition during the life cycle of HIV**. The HIV life cycle generates a number of potential PAMPs (e.g. dsRNA structures, DNA:RNA hybrids, and dsDNA) as well as aberrant localization of molecular structures shared between virus and host (RNA and DNA in endosomes). Some of these are recognized by PRRs and activate expression of antiviral and inflammatory gene products. Recognition of uridine-rich HIV LTR-derived ssRNA and gp120 by TLR7/8 and DC-SIGN, respectively, remain the only experimentally confirmed HIV PAMPs to date.

As illustrated in Figure 4, the viral life cycle is initiated by binding of viral gp120 to the cellular CD4 surface molecule [76]. Such glycoproteins of the viral envelope may be recognized by surface TLRs and CLRs as described for other viruses, such as cytomegalovirus [52-54]. Furthermore, interaction between viral gp41 and the chemokine receptors CXCR4 or CCR5 is required for fusion of the viral envelope with the cellular plasma membrane and release of the viral capsid into the cytoplasm [77-79]. The process of reverse transcription takes place in the cytoplasm, possibly with most viral structures shielded from cellular recognition due to localization in the viral capsid [75]. Intracellularly, ssRNA is recognized by TLR7/8, but given that these receptors are located in the luminal aspect of the endosomal membrane, the viral genome needs to be transported to this compartment, either via viral endocytosis or by autophagy of viral material in the cytoplasm [80]. The two strands of RNA are entwined within the core as a ribonuclear complex with viral proteins forming a dimeric RNA complex [81]. Thus, higher order dsRNA structures represent potential PAMPs for endosomally located TLR3 or cytosolic RLRs, particularly MDA5. Triggering of RIG-I may be prevented by 5'capping of viral genomic RNA, making it similar to mRNA of host origin, and precluding its recognition as foreign [75].

RT is an RNA-dependent DNA polymerase, which uses the viral positive ssRNA genome as template and the virion tRNA as primer for the synthesis of a negative-strand DNA copy [75], thus forming an RNA:DNA hybrid, which may also be recognized by an as yet unidentified receptor. Subsequently, the viral ribonuclease H activity of RT degrades the viral genomic RNA template, except for two resistant purine rich sequences, which then serve as primers for the formation of a complementary DNA plus-strand [75]. Following formation of linear dsDNA, a pre-integration complex consisting of viral DNA and several viral proteins is formed and translocated into the nucleus [82]. This essential step in the HIV replication cycle is mediated by the virion-carried integrase, and once a linear copy of the viral genome has been inserted in the host cellular genome, the integration is for the lifetime of the cell. However, unintegrated circular DNA may persist in the nucleus and be transcribed, particularly in quiescent cells [83]. Therefore, it appears that dsDNA or ssDNA first in the cytoplasm and subsequently in the nucleus may be possible targets for cellular DNA receptors, including TLR9 in endosomes or cytosolic DNA receptors. Indeed, there is recent evidence of cellular mechanisms for recognition and degradation of ssDNA of retroviral origin [72]. Based on data that cytosolic DNA detection activates a potent antiviral response, the IFN-stimulatory DNA response [84], Medzhitov and associates identified an exonuclease named Trex that metabolizes reverse transcribed DNA [72]. In Trex-deficient cells ssDNA derived from endogenous retro-elements accumulates, and mutations in the human Trex gene cause autoimmune manifestations [72]. It is a very intriguing idea, that HIV DNA may be recognized by a host DNA receptor, which however remains to be identified.

Synthesis of new progeny virus is accomplished in a highly regulated manner utilizing host cell enzymes and dependent on cellular or microbial inflammatory or mitotic signals, including the HIV transactivator Tat [75]. Integrated viral DNA is transcribed by host RNA polymerase to produce full-length RNA, which is either integrated into new virions as genomic ssRNA or further processed to produce different mRNAs containing gag, gag-pol, and env sequences. These mRNAs undergo translation, processing, and maturation in the endoplasmic reticulum and Golgi. Gag and gag-pol proteins bind to the plasma membrane containing envelope glycoprotein, and the association of two copies of genomic ssRNA and cellular tRNA molecules finally promote cellular budding and virion release [75]. Genomic RNA, mRNA and various viral structural and regulatory proteins present at this time also represent potential ligands for appropriate cytosolic PRRs. Only after release from the cell, the viral protease mediates cleavage of gag and gag-pol poly-proteins to finally accomplish maturation of the viral core and release of RT, thus completing the life cycle of the virus. The hypothetical possibilities described above for interactions between HIV-derived PAMPs and PRRs are illustrated in Figure 4. Given the fact that PAMPs must be conserved and foreign from self or present in aberrant localizations [51], future research on cellular HIV recognizing PRRs should be focused on the cytoplasm or maybe even the nucleus; and HIV nucleic acids represent good candidates for viral PAMPs.

## Innate immune recognition of HIV

### HIV PAMPs recognized by TLR7/8

Based on the observation that initiation of HAART leads to an almost immediate decline in immune activation, which can be correlated to significant reduction in HIV viraemia, a direct contribution of HIV itself to immune activation has been proposed [85-87]. The first direct link between HIV and innate PRRs was reported in 2004 in a study demonstrating that guanine-uridine-rich ssRNA derived from HIV is recognized by TLR7/8 and stimulates DCs and macrophages to secrete IFN-α and proinflammatory cytokines [56]. A role for TLR7/8 activation in HIV immune activation was supported by studies demonstrating MyD88-dependent activation of plasmacytoid DCs (pDC)s and monocytes by uridine-rich ssRNA sequences from the HIV LTR (ssRNA40) [86]. Moreover, ssRNA40-mediated activation of natural killer (NK) cells has been described, and the activation appears to be critically dependent upon cellular cross-talk between NK cells and CD14+ monocytes [88]. In a study focusing on the requirements for pDC activation, Beignon et al. found that endocytosis followed by viral nucleic acid in the endocytic compartment is required for pDC activation and IFN-α secretion. Although the experimental set-up did not allow for a precise identification of the receptor involved, the data strongly pointed to TLR7, with a possible role for TLR9 [89]. An important strength of this study, however, was the utilization of live virus rather than the less physiological approach involving transfection of synthetic HIV-derived uridine-rich ssRNA. Recently, evidence was presented suggesting that productive infection of DCs requires two distinct HIV-dependent innate signal transduction pathways [90]. It was demonstrated that whereas genomic HIV ssRNA activates TLR8 signalling to NF-κB and initiation of transcription from integrated HIV provirus, interaction between HIV gp120 and DC-SIGN induces Raf-dependent phosphorylation of the NF-κB subunit p65, which is required for elongation of viral transcripts and hence for synthesis of complete viral transcripts and productive infection [90].

Further support for a role of TLR7/8 in HIV immune activation was provided by findings of HIV RNA rendering human lymphoid tissue of tonsillar origin or peripheral blood mononuclear cells (PBMC)s less permissive to HIV replication [91]. In another study, the same authors were able to demonstrate that TLR7/8 stimulation induces changes in the microenvironment unfavourable to HIV, with NK and CD8+ T cells playing an essential role, although no specific soluble factor responsible for these effects was identified [92]. Finally, convincing evidence for the involvement of TLR7/8 triggering in immune activation was provided by histopathological studies in mice, which showed disruption of the lymphoid system, including lymphopenia, abolished antibody production, and alterations in lymphoid microarchitecture resembling HIV-mediated pathology following sustained TLR7 activation [93]. Likewise, repeated CpG DNA administration in mice, activating pDCs through TLR9, resulted in lymphoid pathology, including lymph node hyperplasia, disruption of follicle microarchitecture, and subsequently decreases in numbers of CD4+ and CD8+ T cells, all of which was dependent on type I IFN signalling [94]. As described above, TLR7/8-mediated sensing of uridine-rich HIV RNA, as well as recognition of gp120 by DC-SIGN, represent the only direct evidence of HIV recognition by the innate immune system. This may seem surprising in comparison with other pathogens, which are often recognized by various overlapping families of PRRs. Finally, a recent report of lentivirus vector-induced activation of TLRs suggests that TLR3 may also be involved in sensing of dsRNA structures during HIV infection [95].

### PAMPs from opportunistic pathogens activating TLRs

Activation of innate immune receptors during HIV infection does not only involve PAMPs derived from HIV but also applies to PAMPs originating from opportunistic pathogens and translocated bacteria [38,96]. Considering the wide range of pathogenic microbes that may be present during the course of HIV infection, several TLRs may be involved in microbial recognition and immune activation. Indeed, a study addressing this issue demonstrated that almost all human TLRs can induce CD4+ and CD8+ T cell activation and death, which may contribute to the pathogenesis of immunodeficiency during chronic HIV infection [97].

Almost ten years ago, it was reported that bacterial LPS activates the HIV LTR through TLR4 [98]. This is mediated by NF-κB activation, which induces viral replication due to the presence of NF-κB elements in the HIV LTR [99] (shown in Figure 3 and described in more detail later). Subsequent data on massive bacterial translocation through the damaged GALT during HIV infection suggest that such LPS may trigger TLR4 during chronic immune activation [38]. In a recent clinical study involving HIV-infected patients, it was confirmed that significantly increased LPS levels were associated with chronic HIV infection, and the observed LPS tolerance was diminished in individuals with HIV infection, leading the authors to suggest that HIV infection dysregulates natural TLR responses to subclinical endotoxaemia [100]. Supporting these findings, another study in HIV-infected patients in Guinea Bissau, revealed associations between microbial translocation, measured as plasma LPS concentration, and severity of both HIV-1 and HIV-2 infection [101].

A correlation between bacterial DNA as a measure of bacterial translocation and immune activation in HIV-infected individuals has been demonstrated, and such bacterial DNA may also stimulate innate immune activation through TLR9 or cytosolic DNA receptors [102]. However, despite some authorities arguing for HIV infection to be considered a disease of the gastrointestinal tract [103], several studies question the dominant role assigned to the gastrointestinal mucosa and microbial translocation. For instance, the finding of severe depletion of the GALT in natural hosts of SIV (sooty mangabeys) in the absence of immune activation and immunopathology, may indicate that microbial translocation does not necessarily lead to immune activation [104,105], or at least does not represent an exclusive explanation. At present, it is not clear, whether endotoxaemia directly causes immune activation and CD4+ T cell depletion, or whether it merely reflects a loss of CD4+ T cell host protection and mucosal damage induced by existing immune activation [106].

## Regulation of TLR responsiveness and cell type differences

One important aspect necessary to address when describing interactions between HIV and the innate immune system, is the extensive difference observed between various cell types. Such differences add further complexity to the overall picture, since HIV targets several different cell types, including T cell subsets, monocytes, macrophages, and DCs. Therefore, entirely different recognition mechanisms and immune strategies may exist depending on the cell and tissue involved. Whereas there is solid evidence for TLR7-mediated activation of pDCs, resulting in type I IFN production [86], other cell types appear to be much less sensitive to HIV PAMPs. In primary human macrophages, HIV induces activation independently of TLRs, although infection increases responsiveness to other TLR ligands [107]. This is in agreement with clinical studies, in which TLR expression and responsiveness are increased in viraemic HIV infection [108]. PBMCs from these infected individuals exhibit augmented mRNA expression of TLR2, 3, 4, 6, 7, and 8 as well as increased proinflammatory responsiveness to TLR ligands, suggesting TLR sensitization in chronic HIV infection [108]. It may have major implications that macrophages, which play important roles in transmission and as reservoirs of actively replicating virus, are unable to directly mount an antiviral response towards HIV, but instead become primed to respond to different microbial challenges contributing to immune activation. In this manner macrophages play a key role in inducing and maintaining immune activation in HIV infection [109,110].

Despite TLRs being mainly expressed on cells of the innate immune system, mRNAs encoding TLR 1, 2, 3, 4, 5, 7, and 9 have also been detected in human primary CD4+ T cells, and engagement of specific TLRs trigger secretion of Th1 and Th17 cytokine profiles, suggesting that a subset of TLR ligands can activate resting CD4+ T cells [111-113]. Interestingly, TLR5 stimulation was reported to trigger reactivation of latent HIV provirus from T cells and to activate viral gene expression in central memory T cells [114]. These novel findings underscore the profound cell type differences in HIV-host interactions and also indicate that innate and adaptive immunity should not be regarded as two separate arms but rather as tightly connected and mutually dependent systems.

## Dual role of innate immune activation in HIV infection

### Activation of NF-κB and inflammation

**Figure 5:**
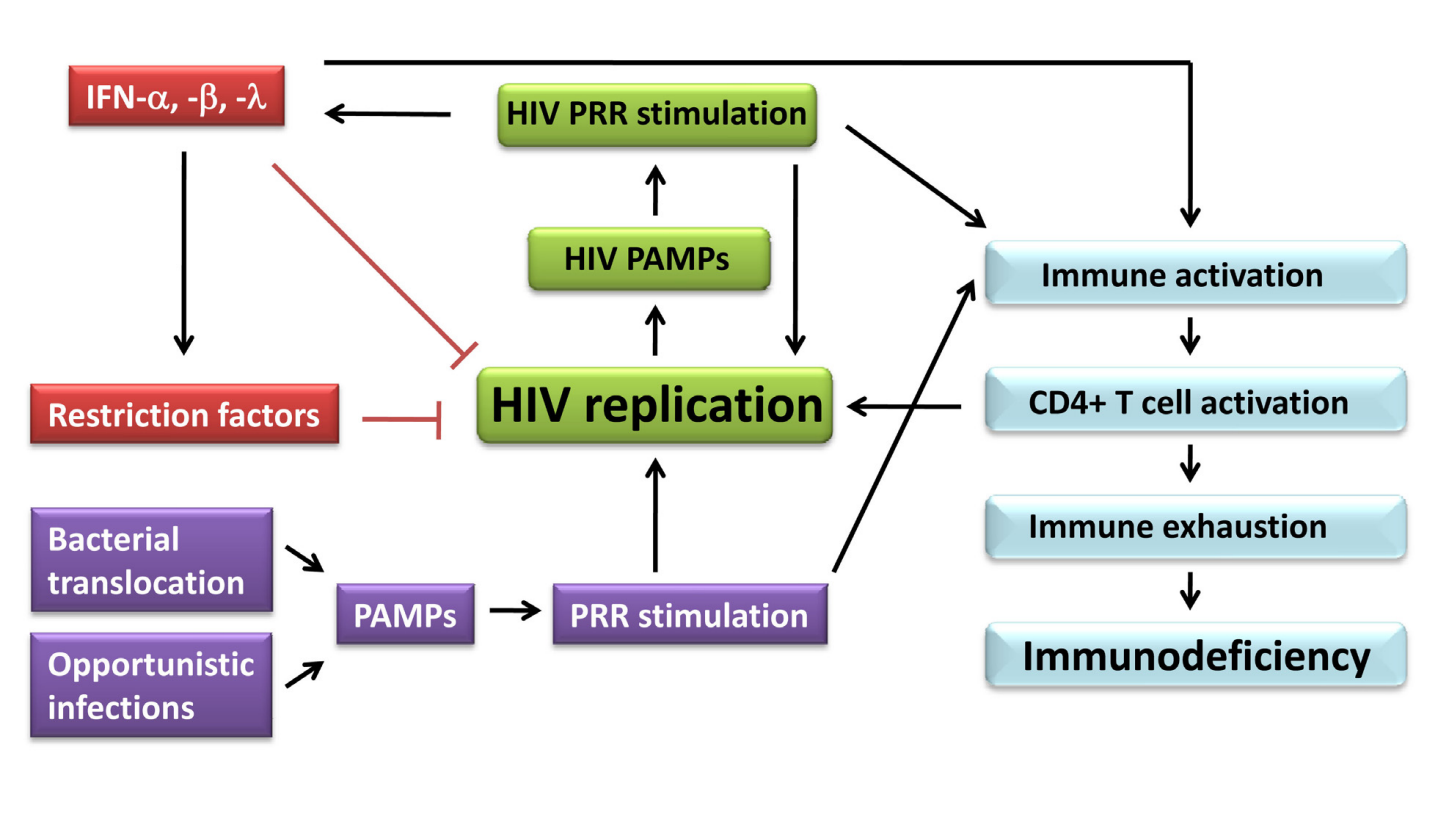
**HIV and innate immune activation - impact on viral control and immunopathology**. HIV infection results in constitutive activation of the innate immune system due to PAMPs derived from HIV, translocated bacteria, or opportunistic pathogens. This stimulates antiviral activities, but also potentially contributes to chronic immune activation. For a more detailed discussion, see text.

The elegant mechanism, by which HIV is capable of exploiting NF-κB to its own advantage to promote viral replication, is a clear example of the ingenuity of HIV. Early studies unravelled that NF-κB perpetuates HIV enhancer activity in infected monocytes, and that κB sites in the HIV LTR are responsible for this phenomenon [99,115]. Moreover, Tat-mediated amplification of HIV transcription in CD4+ T cells was demonstrated to be critically dependent on κB-responsive elements [116]. These findings paved the way for the idea that HIV replication is induced either by the virus itself [117], or alternatively by various opportunistic or translocated pathogens, most of which trigger different classes of immune receptors to activate NF-κB [38]. This is illustrated in Figure 5. The close relationship between immune activation and viral replication is also evidenced by TNF-α-induced NF-κB activation promoting enhanced replication of HIV clade C as compared to other HIV subtypes, which may be explained by the presence of an extra NF-κB element in the HIV clade C LTR promoter [118]. As described above, several lines of evidence strongly suggest that HIV-derived molecules and viral replication are major forces in driving acute and chronic immune activation. This is clearly demonstrated in the reversion of immune activation shortly following initiation of HAART in HIV-infected patients, even before the CD4 count has returned to normal [85]. However, it should be noted, that certain clinical studies examining immunological parameters in elite controllers have revealed some degree of immune activation despite very low or undetectable viral load [119], arguing for non-HIV-derived microbial stimuli as a source of immune activation. In this context, it must be taken into consideration, that circulating levels of virus only poorly reflects the situation in lymphoid or mucosal tissue, in which some degree of viral replication is likely to occur despite undetectable virus in blood.

Innate immune recognition may play a central role in ongoing immune activation through PRR activation, hence resulting in the production of a range of cytokines and chemokines [1,120]. Furthermore, the inflammasome, which is responsible for maturation of pro-IL-1 and -18 to bioactive molecules [121], may also be activated during chronic HIV infection by HIV ligands or danger molecules liberated from damaged tissue, since IL-1 has been linked to HIV-associated dementia, and IL-18 has been suggested to play an important role in the development of progressive immunodeficiency and AIDS [122]. Proinflammatory mediators in turn recruit and activate more immune cells, some of which become infected. In cells with established infection, cytokines, mitogens, and PRR ligands activate further HIV replication via NF-κB, AP-1, and other transcription factors (Figure 3). In this manner, increased viral load may continuously provide new PRR ligands. As illustrated in Figure 5, this may create a scenario, in which a self-perpetuating circle could theoretically drive chronic immune activation. The conceptual problem however remains, that chronic immune activation and CD4+ T cell depletion may amplify each other, therefore making it difficult, if not impossible, to establish which process underlies and drives the other.

### Antiviral and pathological effects of type I IFN

Even prior to the identification of HIV as a human retrovirus causing AIDS, the study of human retroviruses was tightly linked to IFN research [123,124]. One of the hallmarks of a viral infection is the production of type I IFN with antiviral activities [73], including increased degradation of RNA, arrested cell cycle progression, increased antigen presentation, and induction of apoptosis of virus-infected cells [73]. However, IFNs may also exert undesirable effects upon the host, most notably induction of chronic immune activation [125,126] (Figure 5). Therefore, much interest has focused on the role of IFN in HIV pathogenesis [126]. Such IFN may be induced through PRRs either by HIV-derived ligands or PAMPs from opportunistic pathogens.

A pertinent question is whether IFN has any antiviral activities during HIV infection. Several studies have demonstrated that type I IFN does inhibit the replication of HIV in vitro [127-129]. In addition, the recently identified type III IFN (IFN-λ), which exerts antiviral activity mainly at mucosal surfaces [130], has been reported to impair HIV-1 replication in macrophages [131]. The antiviral potency of IFN may however be sensitive to the milieu, as exemplified by a study demonstrating decreased sensitivity of HIV to IFN during conditions of efficient cell-to-cell spread of the virus [132]. Furthermore, type I IFN produced in lymphoid tissue of SIV-infected macaques could not be demonstrated to inhibit viral replication [133]. Studies in natural host of SIV infection have provided further interesting results, since divergent TLR7 and TLR9 signalling and differential type I IFN production was found to distinguish pathogenic and non-pathogenic HIV infections. In sooty mangabeys, which are natural hosts of HIV, only modest immune activation and immunopathology was observed despite high levels of viraemia [47,134]. This has lead to the hypothesis that an attenuated IFN response in sooty mangabeys may enable them to avoid generalized immune activation and therefore may also be desirable in humans during HIV infection [134,135]. In support of this idea, SIV infection triggers a rapid and strong IFN-α response in vivo in both African green monkeys (natural host which do not develop AIDS) and rhesus macaques, but only in African green monkeys is this response efficiently controlled, preventing immune activation and immunodeficiency [48,136]. The view on type I IFN production in natural hosts has recently been broadened, since global genomic analysis and in vivo studies have revealed that acute SIV infection of sooty mangabeys appears to be initially associated with a potent innate immune response, including broad upregulation of IFN-stimulated genes [137], but this immune activation is rapidly resolved [137,138]. These important findings strongly suggest that the modest level of immune activation characteristic of chronic SIV infection is the result of active negative regulatory mechanisms, rather than an intrinsically attenuated innate immune response due to an inability of pDCs to respond to SIV.

In humans, early studies reported a depletion of circulating type I IFN-producing cells in HIV-infected AIDS patients [139] and reduced IFN production from pDCs and PBMCs [140]. However, more recent studies providing evidence of increased pDC frequencies and elevated levels of IFN-α in T cell-rich areas of tonsils from acutely infected and progressing patients, suggest that the observed decrease in circulating pDCs in HIV-infected patients may reflect pDC relocation to lymphoid tissue rather than numerical depletion, supporting a role for IFN in the pathogenesis [125].

Recently, some of the molecular mechanisms, by which IFN-α may cause undesirable immune activation and contribute to disease progression have been delineated. For example, one study demonstrated IFN-α-mediated upregulation of CCR5 on T cell progenitor cells, paradoxically expanding the tropism of CCR5 trophic HIV and potentially accelerating disease progression [141]. Type I IFN has also been demonstrated to suppress the Th17 cell population [142], a phenomenon that has recently been appreciated to play a role in HIV-induced immune dysregulation [143]. Moreover, TLR7-induced IFN-α has been demonstrated to transform pDCs into killer pDCs, resulting in CD4+ T cell apoptosis via the TRAIL pathway [144,145]. A model has been proposed, in which type I IFN-stimulated mechanisms induce death in both HIV-infected and uninfected CD4+ T cells, the latter of which bind HIV without becoming productively infected [126]. In this scenario, type I IFN is beneficial when it kills HIV-infected cells but detrimental when it mediates immunopathogenic apoptosis in uninfected T helper cells. The programmed death (PD)-1 receptor and its ligand (PDL-1) represent another target of IFN-mediated immunopathogenesis. Upregulation of PD-1 expression on HIV-specific T cells is associated with T cell exhaustion and disease progression [146,147], and upregulation of the corresponding ligand PDL-1 has been reported in pDCs upon stimulation with TLR agonists or type I IFN [148].

A more complex picture of the effects of IFN may reconcile the contrasting results in the field. In particular it is important to consider the effects of IFN in the context of infection, including cell type, host, and stage of infection. With increasing insight into the interplay between HIV and IFN, it has become evident that TLR7/8 activation and IFN production may exert opposing effects depending on the stage of infection. Whereas antiviral and antiproliferative effects may be beneficial during acute infection at the expense of a certain degree of immune activation, innate immune activation may be deleterious later during chronic infection. The central question here seems to be, whether the net effect of IFN production is beneficial or harmful to the host, i.e whether positive antiviral effects outweighs the negative consequences of IFN-induced inflammation. In this respect, IFN seems to be a double-edged sword for the organism. Many questions remain to be answered before the full picture of the role of IFN during HIV infection has been clarified.

### Role of DCs, Th17 cells, and regulatory T cells during HIV infection

DCs are of pivotal importance, not only because they are among the earliest targets of HIV, but also due to their ability to capture antigens and initiate T cell responses [149]. Based on differences in function and expression of surface markers, DCs can be divided into several subtypes, among which myeloid DCs (mDC)s are professional antigen presenting cells present in blood, skin and mucosal tissues, whereas pDCs are located in blood and secondary lymphoid organs and play important roles in innate immune responses to viruses through the production of type I IFN [74]. Although mDCs have been reported to play a role in stimulation of HIV-activated adaptive immune responses, it is well documented that mDCs from HIV-infected individuals have reduced capacity to present antigens and stimulate T cells [150,151]. An important discovery concerning the interplay between HIV and DCs was recently reported by Piquet and associates, who described a mechanism by which the HIV envelope protein activates mTOR and S6K signalling, thereby negatively regulating autophagy in DCs and increasing cell-associated HIV and HIV transfer to CD4+ T cells [152]. HIV transmission may also be influenced by TLR signalling. For instance, triggering of TLR2 on DCs increases HIV transmission towards CD4+ T cells, whereas activation of TLR4 reduces virus transmission due to secretion of type I IFNs [153]. Interestingly, opposing roles for mDCs and pDCs in HIV infection have been described [154]. Whereas mDCs enhance HIV infection through capture and subsequent transmission of the virus, pDCs in contrast inhibit HIV replication in T cells through the antiviral activities of IFN-α [154]. In addition, follicular DCs trap and maintain large quantities of HIV during acute HIV infection, thus establishing a viral reservoir in close proximity to susceptible CD4+ T cells in lymphoid tissue [155].

Since pDCs are the major producers of type I IFN, it has been suggested that abnormal migration and localization patterns of this important cell type may be a key in understanding the interplay between HIV and type IFN [126,156]. Besides representing the origin of antiviral type I IFN, pDCs are also one of the main sources of the enzyme indoleamine (2,3)-dioxygenase (IDO), which is involved in tryptophan catabolism and recently described as an important mediator of negative regulation of T cell responses due to tryptophan depletion and accumulation of toxic metabolites. In HIV-infected patients, the rate of tryptophan catabolism is increased and IDO expression is elevated in lymphoid tissues, thereby potentially mediating immunopathology [157].

Within the T cell compartment, much interest has been focused on an altered balance between proinflammatory Th17 cells and regulatory T cells (Treg)s in HIV infection. Th17 cells are CD4+ cells that produce IL-17 and play a central role in immune responses to extracellular bacteria [158]. Initial studies in SIV-infected macaques revealed a reduction in Th17 cells within a few weeks from infection and a negative correlation between plasma virus levels and frequency of Th17 cells [143]. This was followed by Brenchley *et al*., who provided evidence that HIV is capable of infecting Th17 cells in vivo and also demonstrated a significant loss of Th17 cells in the gastrointestinal tract of HIV-infected patients [159]. These findings were confirmed in another study involving natural hosts to SIV, in which pathogenic SIV infection was characterized by selective depletion of Th17 cells and loss of the balance between Th17 cells and Tregs [160]. Studying PBMCs from HIV-infected and -uninfected individuals, it has subsequently been demonstrated that HIV-infected patients display a profound loss of Th17 cells as well as a gradual decline in Tregs during disease progression [161]. These findings were extended by another study reporting on complex perturbations of Th17 subsets during the course of HIV disease [162]. Interestingly, the dysregulated Th17 response during HIV infection may be explained by the reported ability of type I IFN to negatively regulate Th17 development [142]. Thus, sustained expression of type I IFN induced either directly by the virus via TLR7/8 or indirectly by opportunistic viral infections is likely to suppress the Th17 response and hence impair mucosal antibacterial defences and contribute to the chronic enteropathy in HIV infection.

Tregs is a small subpopulation of T cells involved in preventing or inhibiting autoimmune and inflammatory disorders [163], but much controversy exists regarding the role of Tregs in HIV pathogenesis. One study demonstrated expansion of Tregs during HIV infection positively correlating with CD4+ T cell activation and rapid disease progression, indicating a detrimental role of Tregs in the immune control of HIV infection [164]. At the mechanistic level this may be explained by Tregs being major producers of transforming growth factor-β, which promotes tissue fibrosis and limits immune reconstitution [43]. In direct contrast however, several previous studies have reported decreased levels of Tregs in HIV-infected individuals [165], and in one study, depletion of Tregs in HIV infection was found to be associated with immune activation [166]. Collectively, relatively little is known about the precise role of Th17 cells and Tregs in HIV pathogenesis and future studies should shed light on this important issue.

## Innate immune evasion strategies employed by HIV

HIV recognition by PRRs seems to be rather limited, which may indicate that HIV is particularly successful in preventing intimate encounter with the innate immune system. Accumulating evidence suggest that this virus actively avoids recognition by PRRs in order to prevent activation of a proinflammatory and antiviral responses. On a theoretical basis, it can be hypothesized that HIV is in possession of specific strategies to shield or modify its PAMPs within infected cells, for instance by hiding its RNA/DNA in the viral capsid throughout most of the viral life cycle, or by altering its nucleic acids in order not to appear foreign to the host. Below, some of the strategies, by which HIV evades innate immune activation, are described.

Despite the importance of NF-κB for transcription of the viral genome, it may be advantageous for HIV to prevent NF-κB activation in certain situations. In a study addressing the mechanisms of cellular innate immune responses, HIV infection of primary monocyte-derived macrophages did not activate NF-κB [167], indicating that in certain cell types, and in macrophages in particular, HIV inhibits innate immune activation [109]. One report has suggested that TLR4 signalling pathways may be altered during chronic HIV infection, since TLR4-driven NF-κB activation failed to stimulate virus replication, implying that NF-κB alone is insufficient to activate the viral LTR [168]. The mechanism may involve Nef-mediated activation of MAPK phosphatese 1, which negatively regulates TLR4-dependent signalling [169]. In addition, HIV infection of a human myeloid cell line has been found to impair MAPK activation and NF-κB binding to the IL-12 promoter [170]. Likewise exposure of monocyte-derived DCs to recombinant gp120 abrogates LPS-induced IL-12 production [171]. Finally, studies in transgenic Drosophila models have contributed with data showing that vpu inhibits TLR-induced degradation of the IκB homologue Cactus required for NF-κB activation [172], and that Nef interferes with activation of the NF-κB homologue Relish [173].

Evidence is also accumulating with regards to HIV interference with the IFN system. First, direct interaction of HIV gp120 with pDCs inhibits TLR9-mediated responses, including pDC activation, IFN-α secretion, and cytolytic activity of NK cells [174]. Within infected cells, HIV is able to interfere with signal transduction pathways as demonstrated by Doehle *et al*. who observed depletion of IRF3 in HIV-infected CD4+ T cells [175]. IRF3-depletion was dependent on a productive HIV replication cycle and caused disruption of IRF3-mediated signalling pathways, including TLRs and RLRs, in this manner promoting host cell permissiveness for infection with both HIV and opportunistic infections [175]. As to the possible mechanism, HIV accessory proteins Vpr and Vif have been demonstrated to induce IRF-3 degradation [175]. Intriguingly, by specifically targeting IRF3 rather than a TLR signalling molecule located further upstream, HIV is capable of attenuating IRF-dependent immunity while preserving pathways leading to NF-κB activation. The idea that HIV actively suppresses innate immune responses is further supported by studies in primary macrophages, in which HIV infection resulted in a striking absence of IRF3 or IFN gene expression, although the mechanism remains to be determined, since the phenomenon was found to be independent on viral entry, HIV accessory proteins, and reverse transcription [167].

In addition to interfering with the production of IFN, HIV also counteracts the action of IFNs or IFN-inducible proteins. Mammalian cells harbour intrinsic cell-autonomous activities, which can act to suppress viral replication and collectively are referred to as host restriction factors. These host restriction factors are naturally connected to the innate immune response by virtue of their IFN inducibility [176]. Interestingly, HIV accessory proteins are intimately counteracting these antiviral activities to allow viral replication and release [176]. At present, major classes of host restriction factors comprise the APOBEC proteins, TRIM5α, and tetherin, but new proteins with as yet unknown functions are being identified [176]. APOBEC proteins, and in particular APOBEC3G/F, are cytidine deaminases, identified in non-permissive cells, that induce cytidine to uridine editing of negative-sense reverse transcripts resulting in guanosine to adenosine hypermutations in plus-strand cDNA [176-178]. The result of APOBEC function is hypermutation, replication defects, diminished reverse transcription, and ultimately inhibition of viral replication. However, these antiviral mechanisms are counteracted by the viral protein vif, which inhibits APOBEC function by preventing APOBEC packaging in progeny virions by targeting APOBEC3G for proteasomal degradation [179].

TRIM5α belongs to a large family of proteins, several of which are involved in control of viral infections [176]. The action of TRIM5α appears to be dependent upon interaction with cellular cyclophilin A [180]. Subsequently, TRIM5α binds to incoming retroviruses and rapidly recruits them to the proteasome for degradation before significant viral DNA synthesis can occur [181]. TRIM5α mediates early restriction in non-human primates but does not have a significant impact on HIV replication in humans [182]. Presently, it is not known whether HIV counteracts the activity of TRIM5α.

More recently, an IFN-induced restriction factor that prevents retrovirus release from the plasma membrane was identified and named tetherin [183,184]. Tetherin is a glycosylated membrane protein, which results in accumulation of virion particles at the membrane and failure of these particles to be released. The protein exerts antiviral activity by retaining nascent virions on the plasma membrane hence preventing budding of progeny virus particles [183,184]. Tetherin function is counteracted by the HIV membrane protein vpu, thereby securing release of viral progeny [183]. It is not yet clear, exactly how tetherin prevents virus release, but it has been hypothesized that it may form connections between lipid rafts on plasma and viral membranes, thereby physically preventing virus egress [176].

## Genetic polymorphisms influencing HIV infection

An alternative way to gain understanding of the role of innate immune components in the antiviral response and immune activation during HIV infection is through epidemiological studies of genetic polymorphisms in human populations. One of the first studies addressing this question was the description of almost complete protection from HIV infection conferred by homozygosity of a 32 base deletion in CCR5 [185]. Moreover, certain HLA alleles are associated with control of virus replication and slower progression to AIDS, although the underlying mechanism has not been elucidated [186]. Likewise, the mechanism behind the recently demonstrated association between polymorphisms in the inflammasome component NLRP3 and susceptibility to HIV infection remain unexplained but adds to other studies linking inflammasome activation and IL-1/IL-18 production with HIV pathogenesis [122,187].

In the case of TLRs, somewhat more insight into polymorphisms and HIV-induced inflammation exists. One study focused on HAART naïve HIV positive patients from the Swiss HIV cohort, in which Bochud and co-workers reported an association between two single nucleotide polymorphisms (SNP)s in TLR9 and rapid HIV progression as measured by CD4+ T cell decline [188], although the investigators did not evaluate the precise effect of these SNPs on TLR9 signalling. In contrast, a different TLR9 polymorphism has been linked to slow disease progression and found less frequently among individuals with high viral set point [189]. In addition, a frequent functional TLR7 polymorphism resulting in significantly less IFN-α production has been associated with accelerated disease progression and may also be associated with increased HIV susceptibility, since this mutation was present more frequently in patients than in controls [190]. The importance of TLR7 signalling was further supported by a recent article demonstrating sex differences in the TLR7-mediated response of pDCs to HIV [191]. Interestingly, the authors demonstrated that pDCs from women produce markedly more IFN-α in response to HIV-derived TLR7/8 ligands than pDCs from men, resulting in a higher degree of immune activation in women for a given viral load. At the genetic level, this may be explained by the fact that TLR7 is X-linked and therefore women may have higher expression of this receptor due to unbalanced X-inactivation. Clinically, the more robust IFN-α response in women is translated into women exhibiting lower viral loads early in infection but progressing faster to AIDS for any given viral load [192]. Taken together, these studies support the idea of type I IFN having dual functions, including antiviral activities and immune activation.

## Concluding remarks and perspectives

The interactions between HIV and the innate immune system have only recently caught the attention of HIV researchers, and as a consequence remain poorly described. The present picture is that, unlike most other pathogens, innate immune recognition of this virus may not be very elaborate. However, it is still not very well understood, how HIV evades innate immune recognition. This interesting issue points back to the central questions in HIV pathogenesis, as to why the host is unable to recognize and respond adequately to acute HIV infection to prevent the virus from establishing latent viral reservoirs and thereby lifelong chronic infection. Recent insight into this subject indicates that some of the answers should indeed be sought in the interactions between HIV and the innate immune system [193]. The failing early recognition and control of infection by the innate immune system is likely to be of major importance in the pathogenesis of acute HIV infection, allowing establishment of infection and profound damage to innate as well as adaptive immune activities, not least in the GALT. Moreover, the central role played by chronic immune activation is being increasingly appreciated, and innate immune activation may play a pivotal role at this stage of infection. It seems reasonable to assume that PRR-triggered inflammation and type I IFN production induced by HIV or opportunistic pathogens represent ample possibility for initiating and perpetuating this disadvantageous pathological immune activation leading to progressive immunodeficiency. It may be hypothesized that HIV evades innate immune recognition at early stages to establish chronic infection but allows some degree of innate PRR activation at later stages, where immune activation plays a detrimental role for the host. Thus, the mechanisms of innate immune activation may be different in acute versus chronic infection, and elucidating either one may prove to be highly relevant.

As described is this review, surprisingly few innate immune receptors have been implicated in HIV recognition. Eventually, this may be explained by the fact that HIV PAMPs and their respective PRRs still await identification. Alternatively, understanding the mechanisms by which HIV avoids immune recognition by PRRs may provide insight into pivotal aspects of HIV virology and possibly identify molecular targets for therapeutical interference with the viral life cycle. Clearly, the search for innate immune receptors for HIV is still at an early stage, and this interesting subject is likely to lead to answers to central questions in HIV immunopathogenesis. Therefore, an integration of knowledge on the interactions between HIV and both innate and adaptive immunity is a prerequisite for gaining a more profound understanding of HIV immunopathogenesis, and ultimately for applying this knowledge into the development of novel treatment and vaccination strategies to clinical benefit for patients.

## Competing interests

The authors declare that they have no competing interests.

## Authors' contributions

THM was responsible for drafting the manuscript. THM and SRP were responsible for creating Figures 1, 2, 3, 4, and 5. All authors read and approved the final manuscript.
